# Undifferentiated Chordae Tendineae of the Mitral Valve: Large Cohort Study of a Rare Mitral Malformation

**DOI:** 10.3389/fcvm.2021.695536

**Published:** 2021-07-27

**Authors:** Kunjing Pang, Jingjin Wang, Tingting Zhang, Jinlin Wu, Yiming Gao, Yu Liang, Kai Ma, Fengqun Mao, Xiangbin Pan, Shengshou Hu, Shoujun Li

**Affiliations:** ^1^Department of Echocardiography, Fuwai Hospital, National Center for Cardiovascular Diseases, Chinese Academy of Medical Sciences and Peking Union Medical College, Beijing, China; ^2^Department of Cardiac Surgery, Guangdong Academy of Medical Sciences, Guangdong Provincial People's Hospital, Guangzhou, China; ^3^Department of Cardiac Surgery, Fuwai Hospital, National Center for Cardiovascular Diseases, Chinese Academy of Medical Sciences and Peking Union Medical College, Beijing, China

**Keywords:** mitral stenosis, mitral regurgitation, congenital, undifferentiated chordae tendineae, prognosis

## Abstract

**Aims:** This study aimed to investigate the pathology, classification, diagnosis, and surgical prognosis of UCMV.

**Methods and Results:** Consecutive paediatric patients with ≥ moderate-severe mitral regurgitation (MR) and mitral stenosis (MS) were recruited between October 2016 and July 2020. UCMV was diagnosed and classified into three grades according to the involvement of chorda groups and MS presence or absence; other mitral lesions were included as controls. Of 207 eligible patients, 75 with UCMV (10.0 m [interquartile range (IQR): 6.0–21.5]) and 110 with other mitral lesions (16.0 m [IQR: 5.0–43.5]) were diagnosed using echocardiography and surgical exploration. The associated chorda groups of UCMV were confirmed to show high agreement between echocardiography and surgery (kappa = 0.857, *p* < 0.001). At baseline surgery assessment, the UCMV group exhibited worse New York Heart Association functional class, more severe MR and MS grades, and fewer associated complex anomalies (all, *p* < 0.05) than the control group. After a mean follow-up of 8.3 (IQR:2.7–14.4) months and adjustment for covariates, the UCMV group required longer cardiopulmonary bypass and aortic clamp times, but there were no differences in the incidence of adverse events (*p* = 0.584). Class III was associated with higher risk of adverse events than classes I and II (*p* = 0.002).

**Conclusions:** The UCMV spectrum constitutes a primary pathogenesis of paediatric MV dysfunction, which can be optimally diagnosed using echocardiography. Classification based on mitral anatomy and dysfunction can predict the risk of postoperative adverse events.

## Introduction

Congenital mitral valve (MV) lesions in infants and young children are challenging for cardiac surgeons because alternatives, such as mechanical and bioprosthetic valve replacement, are associated with high mortality and morbidity (1–8). The optimal management of MV lesions closely depends on the preoperative assessment of the anatomical subtype. Currently, all recognised congenital MV malformations can be classified into the following nine categories: (1) MV prolapse; (2) functional mitral regurgitation (MR); (3) ischemic MR; (4) isolated cleft; (5) supramitral ring (6) double orifice MV (7) parachute MV; (8) hypoplastic MV, and (9) mitral arcade or hammock MV. The last five types frequently contribute to mitral stenosis (MS). Apart from the mitral arcade, the other eight malformations can be clearly diagnosed using echocardiography (echo) because of their anatomical characteristics (9–14). There has been limited research on the diagnosis of mitral arcade using echo or surgery since Layman proposed it in 1967 (15). Historically, key terms related to it were proposed, such as hammock MV (16), congenital typical MS (6, 17, 18), mitral hemi-arcade (19), and partial hammock MV (20). Each term described one subtype of undifferentiated chordae tendineae. Hammock MV referred to a specific aspect of the mitral arcade when viewed from its atrial side at operation. Congenital typical MS resembled hammock MV combined with MS. Mitral hemi-arcade or partial hammock MV refers to a state where only one chorda group is involved in undifferentiation and another is normal. To comprehensively summarise this series of undeveloped chordae tendineae of the MV, we proposed the concept of the spectrum of undifferentiated chordae tendineae of the MV (UCMV) for the first time. The mitral arcade and its related mitral defects are all included in the spectrum.

This large cohort study aimed to analyse the anatomy, classification, diagnosis, and prognosis of UCMV. By comparing the anatomic description of echo and surgery exploration, we confirmed that UCMV could be optimally diagnosed using echo. We also enrolled patients with other types of congenital MV malformations during the same period as controls to analyse the difference of the surgical prognosis between UCMV and other mitral lesions. The classification of UCMV was designed according to the involvement of chorda groups and the presence or absence of MS (regardless for its severity) and was demonstrated to be advantageous for the prediction of surgical prognosis. This is the first systematic summary of the clinical features and surgical prognosis of UCMV, which was proven to be a dominant pathogenesis of paediatric MV dysfunction.

## Materials and Methods

### Patients

Consecutive inpatient and outpatient infants and children with ≥ moderate-severe MR and ≥ moderate-severe MS from our Paediatric Cardiac Surgery Department were recruited based on echo examination between October 2016 and July 2020. The echo evaluation criteria for MR and MS degree are listed in detail in Supplementary Table 1. The detailed inclusion and exclusion criteria are as follows:

Inclusion criteria: (1) inpatient and outpatient infants and children younger than 18 years of age; (2) echo results met the following criteria for mitral valve dysfunction: (a) only with ≥ moderate-severe MR; (b) or with ≥ moderate-severe MS; (c) or with ≥ moderate-severe MR plus any severity of MS; (d) or with ≥ moderate-severe MS plus any severity of MR.
Exclusion criteria: combined with atrioventricular canal defect, hypoplastic left heart syndrome, single ventricle.

UCMV were diagnosed and classified using echo first. The Surgical indications and plan were discussed by a team of experienced surgeons. The anatomic diagnosis were confirmed through surgical exploration according to the same criteria as echo. All the other MV lesions were treated with the same process as that for MV prolapse, functional MR, isolated mitral valve cleft, ischemic MR, supravalvular mitral ring, parachute MV, and MV hypoplasia. The study was approved by our hospital's committee. We obtained oral parental informed consent (for the echo examination) and written informed consent (for the operation). The study complied with the Declaration of Helsinki.

### UCMV Diagnostic Criteria With Echo and Surgery

**Figure 1:**
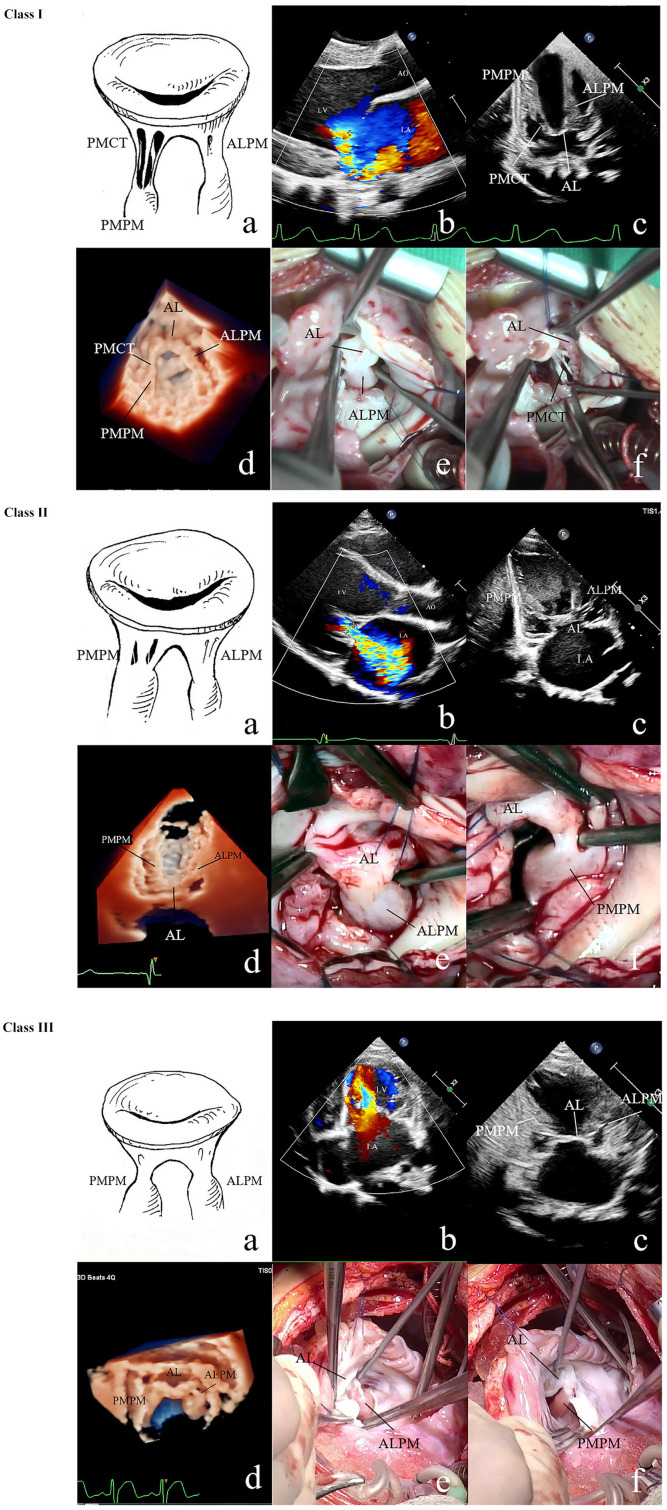
2D and 3D echo and surgical exploration of the three UCMV classes. **Class I**: **(a)** Anatomic diagram of class I of UCMV. Restricted MV insufficiency. The ALCT are undifferentiated, and the ALPM is hypertrophic and elongated and is directly connected with the AL. The PMPM and PMCT are relatively normal. **(b)** The colour Doppler of 2D echo shows restricted severe mitral regurgitation (Carpentier classification: type IIIA). **(c)** 2D echo shows that the ALCT are absent and the ALPM is hypertrophic and elongated and is directly connected with the AL. The PMPM and PMCT are relatively normal. **(d)** 3D echo shows the absent ALCT and the hypertrophic and elongated ALPM being directly attached to the AL, and the PMCT and PMPM being normal. **(e)** Surgical exploration shows that the ALPM is directly connected with the anterior commissure of the AL, and ALCT are absent. **(f)** Surgical exploration shows that the PMCT are relatively normal. **Class II**: **(a)** Anatomic diagram of class II. Restricted MV insufficiency. Both the ALCT and PMCT are undifferentiated. ALPM and PMPM are hypertrophic and elongated and are directly connected with the AL. **(b)** The colour Doppler of 2D echo shows restricted mitral regurgitation. **(c)** 2D echo shows that both the ALCT and PMCT are almost absent, and the ALPM and PMPM, which are hypertrophic and elongated and directly connected with the AL. **(d)** 3D echo shows the hypertrophic and elongated ALPM and PMPM, which are directly connected with the AL. The ALCT and PMCT are absent. **(e)** Surgical exploration shows that the ALCT are absent, and the extremely hypertrophic ALPM are directly attached to the AL. **(f)** Surgical exploration shows that the PMCT are absent, and the extremely hypertrophic PMPM are directly connected with the AL. **Class III**: **(a)** Anatomic diagram of class III. Restricted MV stenosis. Both the ALCT and PMCT are absent. The ALPM and PMPM are hypertrophic and elongated and are directly connected with the AL. **(b)** The colour Doppler of 2D echo shows high speed blood flow over the MV orifice due to MV stenosis (Carpentier classification: predominant valve lesion with normal papillary muscle for MS). **(c)** 2D echo shows that both the ALCT and PMCT are absent. The ALPM and PMPM are hypertrophic and elongated and are directly connected with the AL. **(d)** 3D zoom echo shows hypertrophic and elongated ALPM and PMPM, which are directly connected with the AL. The ALCT and PMCT are absent. **(e)** Surgical exploration shows the hypertrophic ALPM union with the AL. **(f)** Surgical exploration shows the hypertrophic PMPM union with the AL. MV, mitral valve; 2D, two dimensional; 3D, three dimensional; AL, anterior leaflet; ALCT, chordae tendineae attached to the anterolateral papillary muscle; ALPM, anterolateral papillary muscle; PMCT, chordae tendineae attached to the posteromedial papillary muscle; PMPM, posteromedial papillary muscle; UCMV, undifferentiated chordae tendineae of mitral valve.

UCMV could be diagnosed only when the following four criteria were met (Figure 1).

1. Developed MV annulus and leaflets of normal or enlarged size.
2. Two groups of papillary muscles with one being dominant or both being hypertrophic and elongated.
3. Associated chordae tendineae being short or absent, causing the papillary muscles to be closely or directly connected to the leaflets.
4. Restricted motion of the leaflets with limited coaptation Carpentier classification: type IIIA for MR, and predominant valve lesion with normal papillary muscle for MS (17).

### UCMV Classification Criteria With Echo and Surgery

According to the involved chorda groups and the presence or absence of MS, the UCMV were divided into three classes (Figure 1 and Supplementary Table 2):

Class I: Chordae tendineae attached to the anterolateral papillary muscle (ALCT) are mainly associated with un-differentiation; chordae tendineae attached to the posteromedial papillary muscle (PMCT) are mild or normal. MV dysfunction manifest as isolated MR.
Class II: Two groups of chordae are associated with un-differentiation. MV dysfunction manifest as isolated MR.
Class III: Two groups of chordae are associated with un-differentiation. MV dysfunction manifest as mixed MR plus MS (regardless for MS severity) or isolated MS.

### Statistical Analysis

To compare the UCMV group with the control group, continuous variables were tested for normality with the Kolmogorov–Smirnov test and are expressed as mean and standard deviation or median and interquartile range (IQR), as appropriate. Independent *t*-tests were performed for normally distributed variables and Mann–Whitney *U*-tests for non-normally distributed ones. Regarding the comparisons between the different UCMV classifications, analysis of variance was performed for normally distributed variables and the Kruskal–Wallis rank sum test for non-normally distributed ones. Categorical variables are presented as frequencies with percentages and were analysed with the chi-square test or Fisher's exact test, as appropriate. Cohen's or the weighted kappa was used to test the consistency of echocardiographic and surgical parameters. We calculated the freedom from adverse events using the Kaplan–Meier analysis method combined with the log-rank test. Propensity score matching with matching weights was used to balance differences in the baseline status between the UCMV and control groups. All preoperative variables were considered in the matching. The quality of the propensity score matching was assessed by means of a “love plot,” which could graphically display covariate balance before and after adjustment, besides independent sample analysis. A random forest model was constructed to explore the clinical impact of our proposed classification system of UCMV. As a classic algorithm of machine learning, the random forest has high accuracy in disease risk prediction and diagnosis. The sample set was randomly generated by the bootstrapping resampling/bagging method. Five hundred decision trees were constructed in this study, and three variables were randomly selected on each decision tree node. The random forest selected or excluded variables according to their feature importance.

R software (version 3.5.1; R Foundation for Statistical Computing, Vienna, Austria) was used for data analysis. A two-tailed *p* < 0.05 indicated statistical significance.

## Results

### Patients

**Figure 2:**
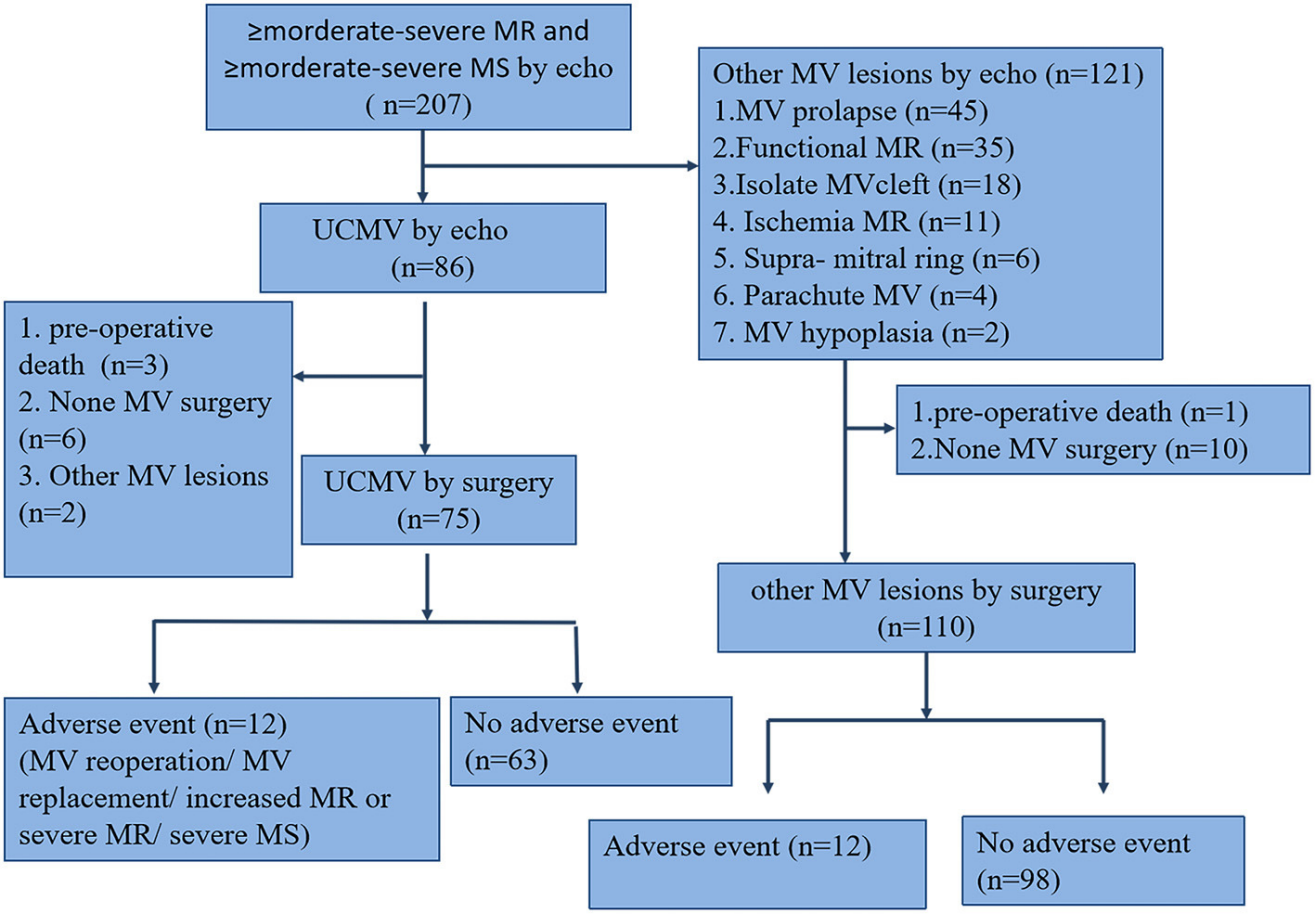
Study population flow chart. The total number of patients with mitral dysfunction was 207. The UCMV and control groups were defined based on mitral valve surgery and echocardiography; other cardiac surgeries and preoperative death were excluded. echo, echocardiography; MV, mitral valve; MR, mitral regurgitation; MS, mitral stenosis; UCMV, undifferentiated chordae tendineae of the mitral valve.

Two hundred and seven paediatric patients with ≥ moderate-severe MV dysfunction diagnosed using echo were included as the eligible cohort. Eighty-six patients with UCMV (41.6%) were diagnosed using echo, of whom 77 underwent mitral valvuloplasty and 75 received UCMV diagnosis confirmation through surgical exploration (40.5%), one patient was diagnosed with MV prolapse, and another with functional MR. One hundred and twenty-one other MV lesions were diagnosed using echo; 110 patients underwent MV valvuloplasty or replacement. The participants' flow chart is shown in Figure 2. The characteristics of the 185 operated cases are summarised in Table 1. The UCMV group exhibited worse New York Heart Association (NYHA) functional class (*p* = 0.031), higher MR (*p* < 0.001) and MS (*p* = 0.014) grade, and fewer complex associated anomalies (*p* < 0.001) than the control group. The clinical data of the three classes of UCMV are shown in Table 2. Apart from MR (*p* = 0.001), MS (*p* < 0.001) grade, and associated chorda groups (*p* < 0.001), class III had worse NYHA functional class than classes I and II (*p* = 0.019). The detailed data of the nine patients with UCMV and eleven patients in the control group who did not undergo operations are showed in online Appendix in Supplementary Material.

**Table 1:** Operative patient characteristics.

	**Level**	**Overall**	**UCMV**	**Control**	***p***
***n***		**185**	**75**	**110**	
**Baseline characteristics**					
Sex (%)	Male	89 (48.1)	35 (46.7)	54 (49.1)	0.862
	Female	96 (51.9)	40 (53.3)	56 (50.9)	
Age (median [IQR])		11.0 [5.0, 35.0]	10.0 [6.0, 21.5]	16.0 [5.0, 43.5]	0.254
Height (median [IQR])		74.0 [66.0, 93.0]	70.0 [66.5, 80.5]	80.0 [65.0, 97.8]	0.099
Weight (median [IQR])		8.4 [6.4, 13.0]	7.7 [6.4, 10.4]	8.9 [6.4, 15.0]	0.098
BSA (median [IQR])		0.4 [0.3, 0.6]	0.4 [0.3, 0.5]	0.4 [0.3, 0.6]	0.113
Cardiothoracic ratio [mean (SD)]		0.6 (0.1)	0.6 (0.1)	0.6 (0.1)	0.158
Oxygen saturation (median [IQR])		99.0 [97.0, 100.0]	98.6 [96.0, 100.0]	99.0 [98.0, 100.0]	0.481
NYHA (%)	I	67 (36.2)	18 (24.0)	49 (44.5)	0.031
	II	81 (43.8)	41 (54.7)	40 (36.4)	
	III	34 (18.4)	15 (20.0)	19 (17.3)	
	IV	3 (1.6)	1 (1.3)	2 (1.8)	
MR grade (%)	0	8 (4.3)	2 (2.7)	6 (5.5)	<0.001
	1	4 (2.2)	2 (2.7)	2 (1.8)	
	2	22 (11.9)	15 (20.0)	7 (6.4)	
	3	57 (30.8)	10 (13.3)	47 (42.7)	
	4	94 (50.8)	46 (61.3)	48 (43.6)	
MS grade (%)	0	159 (85.9)	63 (84.0)	96 (87.3)	0.014
	1	6 (3.2)	2 (2.7)	4 (3.6)	
	2	9 (4.9)	4 (5.3)	5 (4.5)	
	3	5 (2.7)	0 (0.0)	5 (4.5)	
	4	6 (3.2)	6 (8.0)	0 (0.0)	
Associated anomaly (%)	None	47 (25.4)	30 (40.0)	17 (15.5)	<0.001
	Simple	97 (52.4)	39 (52.0)	58 (52.7)	
	Complex	41 (22.2)	6 (8.0)	35 (31.8)	
Previous MV Op (%)	None	172 (93.0)	66 (88.0)	106 (96.4)	0.058
	Yes	13 (7.0)	9 (12.0)	4 (3.6)	
**Operative data**					
CPB time (median [IQR])		100.0 [83.0, 120.0]	111.0 [90.0, 129.0]	99.0 [80.5, 112.8]	0.009
ACC time (median [IQR])		69.0 [50.0, 83.0]	75.0 [60.5, 90.0]	63.0 [48.2, 76.8]	0.007
Ventilation time (median [IQR])		19.0 [9.0, 28.0]	20.0 [9.5, 28.0]	18.0 [8.0, 36.5]	0.462
ICU stay (median [IQR])		96.0 [48.0, 142.0]	95.0 [46.5, 144.0]	99.0 [72.0, 135.2]	0.141
Peritoneal dialysis (%)	None	180 (97.3)	72 (96.0)	108 (98.2)	0.662
	Yes	5 (2.7)	3 (4.0)	2 (1.8)	
Posterior annuloplasty (%)	None	30 (16.2)	6 (8.0)	24 (21.8)	0.021
	Yes	155 (83.8)	69 (92.0)	86 (78.2)	
Leaflet plication (%)	None	108 (58.4)	33 (44.0)	75 (68.2)	0.002
	Yes	77 (41.6)	42 (56.0)	35 (31.8)	
Secondary chorda resection (%)	None	115 (62.2)	43 (57.3)	72 (65.5)	0.335
	Yes	70 (37.8)	32 (42.7)	38 (34.5)	
Papillary muscle splitting (%)	None	118 (63.8)	30 (40.0)	88 (80.0)	<0.001
	Yes	67 (36.2)	45 (60.0)	22 (20.0)	
Chorda detachment (%)	None	155 (83.8)	61 (81.3)	94 (85.5)	0.587
	Yes	30 (16.2)	14 (18.7)	16 (14.5)	
Chorda shortening (%)	None	166 (89.7)	71 (94.7)	95 (86.4)	0.114
	Yes	19 (10.3)	4 (5.3)	15 (13.6)	
Leaflet patch augmentation (%)	None	176 (95.1)	67 (89.3)	109 (99.1)	0.007
	Yes	9 (4.9)	8 (10.7)	1 (0.9)	
Leaflet resection (%)	None	183 (98.9)	73 (97.3)	110 (100.0)	0.318
	Yes	2 (1.1)	2 (2.7)	0 (0.0)	
Leaflet cleft closure (%)	None	168 (90.8)	74 (98.7)	94 (85.5)	0.005
	Yes	17 (9.2)	1 (1.3)	16 (14.5)	
Supramitral ring resection (%)	None	171 (92.4)	71 (94.7)	100 (90.9)	0.506
	Yes	14 (7.6)	4 (5.3)	10 (9.1)	
**Outcomes**
AE (%)	None	161 (87.0)	63 (84.0)	98 (89.1)	0.43
	Yes	24 (13.0)	12 (16.0)	12 (10.9)	
AE-MR (%)	None	178 (96.2)	70 (93.3)	108 (98.2)	0.192
	Yes	7 (3.8)	5 (6.7)	2 (1.8)	
AE-MS (%)	None	176 (95.1)	71 (94.7)	105 (95.5)	1
	Yes	9 (4.9)	4 (5.3)	5 (4.5)	
AE-MVR (%)	None	180 (97.3)	72 (96.0)	108 (98.2)	0.662
	Yes	5 (2.7)	3 (4.0)	2 (1.8)	
AE-ReOp (%)	None	175 (94.6)	70 (93.3)	105 (95.5)	0.768
	Yes	10 (5.4)	5 (6.7)	5 (4.5)	
Repeated on-pump (%)	None	169 (91.4)	66 (88.0)	103 (93.6)	0.283
	Yes	16 (8.6)	9 (12.0)	7 (6.4)	
FU time (median [IQR])		8.3 [2.7, 14.4]	9.9 [3.2, 16.4]	7.0 [2.5, 13.9]	0.362

*Results are given as the number (percent) or the mean ± SD or median (interquartile range). Simple anomaly: ventricle septal defect, atrial septal defect; patent ductus arteriosus*.

*Complex anomaly: aortic valve stenosis, aortic arch interruption; co-arctation of the aorta; sub-aortic valve stenosis; hypertrophic cardiomyopathy, Tetralogy of Fallot*.

*UCMV, undifferentiated chordae tendineae of the mitral valve; MV, mitral valve; BSA, body surface area; SD, standard deviation; IQR, interquartile range; MR, mitral regurgitation; MS, mitral stenosis; NYHA, New York Heart Association; AE, adverse event; AE-MR, mitral regurgitation increase or severe mitral regurgitation; AE-MS, severe mitral stenosis, AE-MVR, mitral replacement; AE-ReOp, mitral re-operation; OR, operation; ACC, aortic clamping; CPB, cardiopulmonary bypass; ICU, intensive care unit; FU, follow-up*.

**Table 2:** Characteristics of the three classes of UCMV.

	**Level**	**Overall**	**I**	**II**	**III**	***p***
***n***		**75**	**27**	**36**	**12**	
**Baseline characteristics**						
Sex (%)	Male	35 (46.7)	12 (44.4)	17 (47.2)	6 (50.0)	0.946
	Female	40 (53.3)	15 (55.6)	19 (52.8)	6 (50.0)	
Age (median [IQR])		10.0 [6.0, 21.5]	10.0 [6.5, 24.5]	10.0 [5.8, 19.2]	13.0 [7.0, 22.8]	0.788
Height (median [IQR])		70.0 [66.5, 80.5]	71.0 [67.5, 80.5]	70.5 [66.0, 80.0]	70.0 [69.0, 81.5]	0.901
Weight (median [IQR])		7.7 [6.4, 10.4]	7.7 [6.6, 10.6]	7.4 [6.1, 10.1]	8.3 [7.3, 10.5]	0.713
BSA (median [IQR])		0.4 [0.3, 0.5]	0.4 [0.3, 0.5]	0.4 [0.3, 0.5]	0.4 [0.4, 0.5]	0.82
Cardiothoracic ratio [mean (SD)]		0.6 (0.1)	0.6 (0.1)	0.6 (0.1)	0.6 (0.1)	0.585
Oxygen saturation (median [IQR])		98.6 [96.0, 100.0]	98.0 [96.5, 100.0]	98.3 [96.0, 100.0]	99.0 [97.8, 99.2]	0.999
NYHA (%)	I	18 (24.0)	11 (40.7)	7 (19.4)	0 (0.0)	0.019
	II	41 (54.7)	14 (51.9)	21 (58.3)	6 (50.0)	
	III	15 (20.0)	2 (7.4)	7 (19.4)	6 (50.0)	
	IV	1 (1.3)	0 (0.0)	1 (2.8)	0 (0.0)	
MR grade (%)	0	2 (2.7)	0 (0.0)	0 (0.0)	2 (16.7)	0.001
	1	2 (2.7)	0 (0.0)	0 (0.0)	2 (16.7)	
	2	15 (20.0)	8 (29.6)	4 (11.1)	3 (25.0)	
	3	10 (13.3)	2 (7.4)	7 (19.4)	1 (8.3)	
	4	46 (61.3)	17 (63.0)	25 (69.4)	4 (33.3)	
MS grade (%)	0	63 (84.0)	27 (100.0)	36 (100.0)	0 (0.0)	<0.001
	1	2 (2.7)	0 (0.0)	0 (0.0)	2 (16.7)	
	2	4 (5.3)	0 (0.0)	0 (0.0)	4 (33.3)	
	4	6 (8.0)	0 (0.0)	0 (0.0)	6 (50.0)	
Combined with anomaly (%)	None	30 (40.0)	10 (37.0)	13 (36.1)	7 (58.3)	0.189
	Simple	39 (52.0)	14 (51.9)	22 (61.1)	3 (25.0)	
	Complex	6 (8.0)	3 (11.1)	1 (2.8)	2 (16.7)	
Previous MV Op (%)	None	66 (88.0)	23 (85.2)	32 (88.9)	11 (91.7)	0.826
	Yes	9 (12.0)	4 (14.8)	4 (11.1)	1 (8.3)	
OR-associated chorda groups (%)	None	26 (34.7)	26 (96.3)	0 (0.0)	0 (0.0)	<0.001
	Yes	49 (65.3)	1 (3.7)	36 (100.0)	12 (100.0)	
OR-ALPM (%)	Involved	75 (100.0)	27 (100.0)	36 (100.0)	12 (100.0)	NA
OR-PMPM (%)	None	28 (37.3)	27 (100.0)	1 (2.8)	0 (0.0)	<0.001
	Involved	47 (62.7)	0 (0.0)	35 (97.2)	12 (100.0)	
OR-ALCT (%)	Short	50 (66.7)	18 (66.7)	28 (77.8)	4 (33.3)	0.018
	Absent	25 (33.3)	9 (33.3)	8 (22.2)	8 (66.7)	
OR-PMCT (%)	Normal	27 (36.0)	27 (100.0)	0 (0.0)	0 (0.0)	<0.001
	Short	34 (45.3)	0 (0.0)	30 (83.3)	4 (33.3)	
	Absent	14 (18.7)	0 (0.0)	6 (16.7)	8 (66.7)	
**Operative data**						
CPB time (median [IQR])		111.0 [90.0, 129.0]	100.0 [80.5, 119.0]	116.0 [93.2, 127.2]	123.0 [106.2, 146.8]	0.167
ACC time (median [IQR])		75.0 [60.5, 90.0]	76.0 [55.0, 85.0]	75.5 [59.5, 90.0]	72.5 [68.8, 115.8]	0.581
Posterior annuloplasty (%)	None	6 (8.0)	1 (3.7)	1 (2.8)	4 (33.3)	0.002
	Yes	69 (92.0)	26 (96.3)	35 (97.2)	8 (66.7)	
Leaflet plication (%)	None	33 (44.0)	11 (40.7)	10 (27.8)	12 (100.0)	<0.001
	Yes	42 (56.0)	16 (59.3)	26 (72.2)	0 (0.0)	
Secondary chordae resection (%)	None	43 (57.3)	16 (59.3)	17 (47.2)	10 (83.3)	0.088
	Yes	32 (42.7)	11 (40.7)	19 (52.8)	2 (16.7)	
Papillary muscle splitting (%)	None	30 (40.0)	16 (59.3)	12 (33.3)	2 (16.7)	0.023
	Yes	45 (60.0)	11 (40.7)	24 (66.7)	10 (83.3)	
Chordae detachment (%)	None	61 (81.3)	24 (88.9)	29 (80.6)	8 (66.7)	0.255
	Yes	14 (18.7)	3 (11.1)	7 (19.4)	4 (33.3)	
Chordae shortening (%)	None	71 (94.7)	26 (96.3)	33 (91.7)	12 (100.0)	0.482
	Yes	4 (5.3)	1 (3.7)	3 (8.3)	0 (0.0)	
Leaflet patch augmentation (%)	None	67 (89.3)	23 (85.2)	34 (94.4)	10 (83.3)	0.381
	Yes	8 (10.7)	4 (14.8)	2 (5.6)	2 (16.7)	
Leaflet resection (%)	None	73 (97.3)	26 (96.3)	35 (97.2)	12 (100.0)	0.802
	Yes	2 (2.7)	1 (3.7)	1 (2.8)	0 (0.0)	
Leaflet cleft closure (%)	None	74 (98.7)	27 (100.0)	35 (97.2)	12 (100.0)	0.578
	Yes	1 (1.3)	0 (0.0)	1 (2.8)	0 (0.0)	
Supramitral ring resection (%)	None	71 (94.7)	26 (96.3)	35 (97.2)	10 (83.3)	0.16
	Yes	4 (5.3)	1 (3.7)	1 (2.8)	2 (16.7)	
**Outcomes**						
AE (%)	None	63 (84.0)	24 (88.9)	33 (91.7)	6 (50.0)	0.002
	Yes	12 (16.0)	3 (11.1)	3 (8.3)	6 (50.0)	
AE-MR (%)	None	70 (93.3)	24 (88.9)	35 (97.2)	11 (91.7)	0.409
	Yes	5 (6.7)	3 (11.1)	1 (2.8)	1 (8.3)	
AE-MS (%)	None	71 (94.7)	27 (100.0)	36 (100.0)	8 (66.7)	<0.001
	Yes	4 (5.3)	0 (0.0)	0 (0.0)	4 (33.3)	
AE-MVR (%)	None	72 (96.0)	27 (100.0)	34 (94.4)	11 (91.7)	0.379
	Yes	3 (4.0)	0 (0.0)	2 (5.6)	1 (8.3)	
AE-ReOp (%)	None	70 (93.3)	25 (92.6)	34 (94.4)	11 (91.7)	0.928
	Yes	5 (6.7)	2 (7.4)	2 (5.6)	1 (8.3)	
Repeated on-pump (%)	None	66 (88.0)	24 (88.9)	34 (94.4)	8 (66.7)	0.037
	Yes	9 (12.0)	3 (11.1)	2 (5.6)	4 (33.3)	
Ventilation-time (median [IQR])		20.0 [9.5, 28.0]	20.0 [10.0, 28.0]	21.5 [8.0, 48.8]	19.0 [10.0, 25.0]	0.969
ICU stay (median [IQR])		95.0 [46.5, 144.0]	96.0 [36.0, 145.5]	96.0 [48.0, 135.8]	48.5 [47.2, 103.6]	0.534
Peritoneal dialysis (%)	None	72 (96.0)	27 (100.0)	35 (97.2)	10 (83.3)	0.043
	Yes	3 (4.0)	0 (0.0)	1 (2.8)	2 (16.7)	
FU time (median [IQR])		9.9 [3.2, 16.4]	12.6 [8.5, 16.4]	8.0 [2.2, 14.1]	6.6 [2.6, 16.7]	0.251

*Results are given as the number (percent) or the mean ± SD or median (interquartile range)*.

*UCMV, undifferentiated chordae tendineae of the mitral valve; MV, mitral valve; BSA, body surface area; SD, standard deviation; IQR, interquartile range; MR, mitral regurgitation; MS, mitral stenosis; NYHA, New York Heart Association; AE, adverse event; AE-MR, mitral regurgitation increase or severe mitral regurgitation; AE-MS, severe mitral stenosis; AE-MVR, mitral replacement; AE-ReOp, mitral re-operation; OR, operation; ALPM, antero-lateral papillary muscle; ALCT, chordae attached to the ALPM; PMPM, posteromedial papillary muscle; PMCT, chordae attached to the PMPM; ACC, aortic clamping; CPB, cardiopulmonary bypass; ICU, intensive care unit; FU, follow-up*.

### Echo and Surgical Exploration

The anatomy of the ALCT or PMCT of the 75 patients with UCMV was described as short, absent, or normal using both echo and surgical exploration. If the tip of the papillary muscle was just beneath the leaflet, the chordae connected to it would be diagnosed as short. If the tip of the papillary muscle was directly attached to the leaflets, chordae absence would be diagnosed. The short and normal chordae are shown in Supplementary Figure 1. Absent chordae are shown in Figure 1. All anatomic data of the mitral tensor apparatus are listed in Table 2. The associated chorda groups were diagnosed with good agreement between echo and surgery (kappa = 0.857, *p* < 0.001; Supplementary Table 3).

### Follow-Up

**Figure 3:**
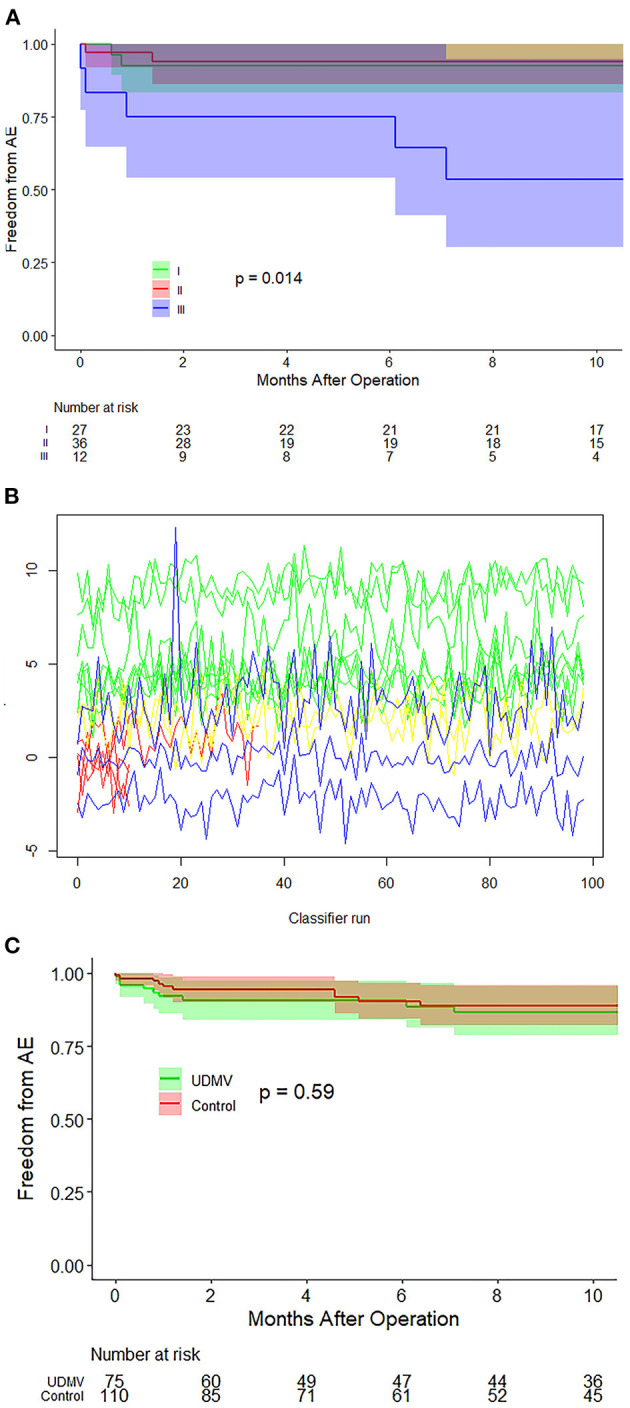
Survival curves and random forest model. **(A)** Survival curves of the three classes of UCMV show that class III is associated with a higher incidence of adverse events. **(B)** The random forest model proved the classification to be of great importance in the prediction of adverse event risk. **(C)** Survival curves of the UCMV and control groups show that there is no significant difference in the surgical prognosis between the UCMV and control groups (*p* = 0. 59). UCMV, undifferentiated chordae tendineae of the mitral valve.

All patients were clinically followed up for 8.3 (IQR: 2.7–14.4) months; echo, electrocardiographic, and radiographic examinations were completed in the clinic. The patients with class III of UCMV required repeated on-pumps (*p* = 0.037) and peritoneal dialysis (*p* = 0.043) to a greater extent than those in classes I–II (Table 2). No postoperative death occurred. MR increase or severe MR, re-mitral operation, MV replacement (MVR), and severe MS were identified as adverse events. Twelve of 75 patients with UCMV developed adverse events, including three MR increases (1 plus re-operation) in class I, one MR increase + one severe MS + two MVRs (plus re-operation) in class II, and four severe MSs (1 plus severe MR) + one Ross II MVR (plus re-operation) in class III. The incidence of adverse events of the three classes was significantly different (*p* = 0.002) (Table 2). *Post-hoc* comparison showed that there were significant difference in class III vs. class I (*p* = 0.042) and class III vs. class II (*p* = 0.013), but no significant difference in class I vs. class II (*p* = 1.000). The Kaplan–Meier survival curve also showed that class III was associated with a higher incidence of adverse events (*p* = 0.014, Figure 3A). The random forest model showed the classification to be of great advantage for the diagnosis and risk prediction of the risk of postoperative adverse events (Figure 3B).

In the control group, adverse events occurred in 12 of 110 patients, including one MR increase + one MVR +one re-operation in the ischemia MR group, one severe MR in the supramitral ring group (plus re-operation), four severe MS in the parachute MV group, one severe MS in the hypoplasia MV group, and one MVR (plus re-operation) and two re-operation in the mitral prolapse group. To balance differences in the baseline status between the UCMV and the control group, all preoperative variables were considered in the propensity score matching, including demographics, NYHA class, MR grade, MS grade, and associated anomalies (Supplementary Table 4 and Supplementary Figure 2). After adjustment, the UCMV group required a longer cardiopulmonary bypass time (*p* = 0.011) and aortic clamping time (*p* = 0.007) than the control group but presented no significant difference in ventilation time (*p* = 0.779), intensive care unit stay time (*p* = 0.129), and incidence of adverse events (*p* = 0.584) (Supplementary Table 4). The Kaplan–Meier survival curve also showed that there was no significant difference in the risks for the adverse events between the UCMV and control group (*p* = 0.59) (Figure 3C).

The detailed data of patients who didn't receive operations are listed in online Appendix in Supplementary Material.

## Discussion

The spectrum of UCMV comprises mitral arcade and other mitral defects related to it (Supplementary Table 2). The main anatomy of mitral arcade involves the connexion of the left ventricular papillary muscles to the anterior leaflet of the MV being either direct or through the interposition of unusually short chordae. The embryologic aetiology is believed to be the result of an arrest in the developmental stage of MV before attenuation and lengthening of the collagenised chordae (15). However, some of our cases did not fit the classic mitral arcade pattern but comprised incomplete forms and appeared in a spectrum of undifferentiated chordae tendineae. Some mitral hemi-arcade and partial hammock MV have been reported in recent years (19, 20), and some reported adult cases (21–25) support these findings. Accordingly, we proposed the concept of a spectrum of UCMV and classified it into three grades according to the involved chorda groups and presence or absence of MS. In the spectrum, the severity of lesions from class I to class III were progressing from mild to severe, signifying that mitral chorda developmental arrest occurs increasingly earlier from class I to class III, and therefore, class III has the worst MV dysfunction and surgical prognosis (Figures 3A,B). The anatomy of classic mitral arcade is similar to that of class III and a part of class II. The arcade is formed by two groups of hypertrophic papillary muscles and a fibre bridge, composed of the lower thickening edge of the anterior mitral leaflet and the intervening chordae (15). The fibre bridge was not typical in some of our patients, especially in class I and part of class II patients. Thus, we did not list it as one of the diagnostic criteria for UCMV. Typically congenital MS belongs to class III of UCMV with two groups of chordae being undifferentiated and MS presence (17). Another notable anatomic feature of UCMV is that the ALCT was entirely associated with un-differentiation, and no isolated PMCT was found, possibly because the attenuation and lengthening of the PMCT is completed earlier than that of the ALCT. The reported mitral hemi-arcade and partial hammock MV showed the same anatomical features (19, 20). The anatomy of class I of UCMV and that of the parachute-like asymmetric MV proposed by Oosthoek (23) are extremely similar. The elongated anterolateral papillary muscle is connected with the leaflet directly or through the unusually short chordae, and the posteromedial papillary muscle is normal. The anatomy of undifferentiated papillary muscles proposed by Matsumaru (24) is also same as that of parachute-like asymmetric MV. Accordingly, we may hypothesise that the parachute-like asymmetric MV, undifferentiated papillary muscles and class I of UCMV should comprise the same substrate of UCMV. UCMV constituted the dominant proportion of all eight types of MV defects (41.6% diagnosed using echo and 40.5% diagnosed through surgery), Similar results have been obtained from other studies (6, 17, 18). Consequently, we can conclude that UCMV is a primary pathogenesis of paediatric MV dysfunction.

A high level of accuracy can be achieved when investigating the UCMV anatomy using echo (Supplementary Table 3). Abnormally hypertrophic and elongated papillary muscles can be more easily imaged with the 3D full-volume or 3D zoom mode of transthoracic echo. It is important to distinguish the separate muscular chordae of some normal MV from UCMV (26), which have fully developed chordae. ALCT dysplasia in class I is more common in children than previously reported, and many patients do not develop noticeable mitral insufficiency during childhood, and remain undiagnosed until they reach adulthood (19–21). In some patients of our cohort, ≥ moderate to severe MR during childhood was related to comorbid defects leading to increased left ventricular preload (Table 2). The mechanism of UCMV dysfunction involves extremely short chordae or hypertrophic papillary muscles (union with leaflet), reducing the interchordal spaces and leading to abnormal excursion of the leaflets (17). As a result, the motion of the leaflets is restricted with limited coaptation leading to MR or MS. In our operative inspections, several patients were found to have “bare” parts of leaflets without chordae attached to them (Supplementary Figure 3), which might be another pathological feature of UCMV. The “bare” parts of the leaflets can lead to poor alignment and exacerbate MR. Unfortunately, we did not satisfactorily identify these leaflets using echo because of difficulties in imaging all chordae. Most MV annuli of class I and II were enlarged, and the leaflets were thickened, while these features were also common in other MR defects in the control group. Four patients in the class III group had supravalvular rings and two patients had commissure unions; all of them had fully developed annuli of sufficient size, which should be distinguished from MV hypoplasia.

Though the structure of UCMV is more complex, the same short to midterm postoperative prognosis as of the other MV lesions can be achieved through tailored MV repair techniques (Figure 3C and Supplementary Table 4). Our precise anatomic diagnosis and classification of UCMV with echo especially facilitated the development of precise techniques for MV repair (23–25). The main surgical techniques for the UCMV cohort included chorda detachment, papillary muscle splitting or detachment, leaflet plication, and posterior annuloplasty (27) (Table 2). Favourable short to midterm outcomes were achieved by most patients in the class I and class II groups, of whom 84.2% lived asymptomatically without > moderate MR or MS. It was difficult to relieve the MS of class III; only 50.0% of patients were found to be free from adverse events postoperatively (Table 2). This result was similar to that of the control group (Table 1) in which 42.9% of patients with MS developed surgical adverse events. It could therefore be said that severe MS is the most challenging condition for paediatric cardiac surgeons.

## Study Limitations

Our cohort was limited to paediatric patients with ≥ moderate to severe MR and ≥ moderate to severe MS; some cases of mild MV dysfunction and adult patients with UCMV were excluded, which precluded completely reflecting the distribution of the UCMV spectrum. There were three patients with double orifice MV who were excluded because their MV dysfunction did not meet the “moderate to severe degree” criterion. Therefore, our control group lacked double orifice MV. The follow-up was not sufficiently long for non-significant differences between the UCMV and control groups to be clearly determined. The final rate of AE of UCMV and control group could be affected to some extend, as some AE are associated with degenerative changes, more than acute, post-surgical events. Although we used a propensity score matching approach, the inhomogeneous pathophysiology of the control group may represent a confounding factor with regard to the aetiology of MS/MR.

## Conclusions

We put forward and systematically characterised the anatomy, diagnosis, classification, and surgical prognosis of UCMV, which should be the most common pathogenesis of paediatric MV dysfunction. Mitral arcade or hammock MV, typically congenital MS, parachute-like asymmetric MV, and hemi-arcade MV are all included in the spectrum. The diagnosis and classification criteria of UCMV were proven advantageous for the improvement of MV repair techniques and prediction of postoperative prognosis. The class III has the worst MV dysfunction and surgical prognosis. The elucidation of the predominant mechanism underlying mitral lesions in infants and children represents a significant step forward in addressing the MV conundrums in paediatric cardiac surgery.

## Data Availability Statement

The raw data supporting the conclusions of this article will be made available by the authors, without undue reservation.

## Ethics Statement

The studies involving human participants were reviewed and approved by Fuwai Hospital, National Center for Cardiovascular Diseases, Chinese Academy of Medical Sciences and Peking Union Medical College. Written informed consent to participate in this study was provided by the participants' legal guardian/next of kin.

## Author Contributions

KP and SL conceived and designed the study. JWa, TZ, YG, YL, KM and FM performed data extraction. JWu conducted statistical analysis. KP wrote the first draft of the manuscript. XP and SH reviewed, interpreted, and commented on the final version. All authors contributed to the article and approved the submitted version.

## Conflict of Interest

The authors declare that the research was conducted in the absence of any commercial or financial relationships that could be construed as a potential conflict of interest.

## Publisher's Note

All claims expressed in this article are solely those of the authors and do not necessarily represent those of their affiliated organizations, or those of the publisher, the editors and the reviewers. Any product that may be evaluated in this article, or claim that may be made by its manufacturer, is not guaranteed or endorsed by the publisher.

## Supplementary Material

Supplementary Figure 1. Class I: (b) The colour Doppler of two-dimensional (2D) echo shows restricted severe mitral regurgitation. **(c)** 2D echo shows that the anterolateral chordae tendineae (ALCT) are absent and the anterolateral papillary muscle (ALPM) is hypertrophic and elongated and is directly connected with the anterior leaflet (AL). The posteromedial papillary muscle (PMPM) and chordae tendineae (PMCT) are relatively normal. **(d)** Three-dimensional (3D) echo shows the absent ALCT and the hypertrophic and elongated ALPM being directly attached to the AL, with the PMCT and PMPM being normal. **(e)** Surgical exploration shows that the ALPM is directly connected with the anterior commissure of the AL, and ALCT are absent. **(f)** Surgical exploration shows that the PMCT are relatively normal. **Class II: (b)** The colour Doppler of 2D echo shows restricted mitral regurgitation. **(c)** 2D echo shows that both the ALCT and PMCT are almost absent, and the ALPM and PMPM are hypertrophic and elongated and directly connected with the AL. **(d)** 3D echo shows the extremely hypertrophic and elongated ALPM and PMPM, which are directly connected with the AL. The ALCT and PMCT are absent. **(e)** Surgical exploration shows that the ALCT are absent, and the extremely hypertrophic ALPM are directly attached to the AL. **(f)** Surgical exploration shows that the PMCT are absent, and the extremely hypertrophic PMPM are directly connected with the AL. **Class III: (b)** The colour Doppler of 2D echo shows mitral stenosis plus mitral regurgitation. **(c)** 2D echo shows that both the ALCT and PMCT are absent. The ALPM and PMPM are hypertrophic and elongated and directly connected with the AL. **(d)** 3D zoom echo shows hypertrophic and elongated ALPM and PMPM, which are directly connected with the AL. The ALCT and PMCT are absent. **(e)** Surgical exploration shows the hypertrophic ALPM union with the AL. **(f)** Surgical exploration shows the hypertrophic PMPM union with the AL.
