# Supplementary material for: Undifferentiated Chordae Tendineae of the Mitral Valve: Large Cohort Study of a Rare Mitral Malformation
Source: Front Cardiovasc Med. 2021 Jul 27;8:695536. doi: 10.3389/fcvm.2021.695536 (PMC8353112; doi:10.3389/fcvm.2021.695536)
Supplement: Supplementary file 5 [file Data_Sheet_1.DOC]

**Online Appendix**

**Echocardiographic grading severity of mitral regurgitation (MR) in infants and children**

With reference to the native valvular regurgitation guidelines for adults by the American Society of echocardiography in 2017 (**14**), we stipulated the evaluation criteria for MR in infants and children. **Online Table 1** shows all six reference indexes; mild or severe MR can be diagnosed if four of the six indexes in Online Table 1 are met. If not, the proximal isovelocity surface area method was required to quantitatively measure the effective regurgitation orifice area index to further judge the degree of MR.

**Online Table 1. Echocardiographic grading severity of MR in infants and children**

| **MR severity** | | | | |
| --- | --- | --- | --- | --- |
|  | Mild | Moderate | | Severe |
| **MR class** | 1 | 2 | 3 | 4 |
| **LV size (with normal LV function)** | Normal | Normal or mildly dilated | | Dilated |
| **Colour flow jet area** | Small, central, narrow, often brief | Variable | | Large central jet (>50% of the LA) or eccentric wall-impinging jet of variable size |
| **Flow convergence** | Not visible, transient, or small | Intermediate in size and duration | | Large throughout systole |
| **CWD jet** | Faint, partial, parabolic | Dense but partial or parabolic | | Holosystolic /dense/triangular |
| **VCW (cm)** | <0.25 | 0.25–0.37 | 0.38–0.50 | ≥0.50 |
| **EROAI (cm^2^/m^2^)** | <0.12 | 0.12–0.17 | 0.18–0.24 | ≥0.25 |

LV= left ventricle, LA= left atrium, CWD = continuous wave Doppler, EROA= effective regurgitation orifice area, BSA= body surface area, EROAI = effective regurgitation orifice area index, equal to EROA/BSA, MR = mitral regurgitation, VCW = vena contracta width.

**Echocardiographic evaluation criteria for mitral stenosis (MS)**

We referred to the mean Doppler-derived transmitral pressure gradients (MPG) as follows: 6≤MPG<8, mild MS (grade 1) could be diagnosed, 8≤MPG<10 was judged as moderate MS (grade 2), 10≤MPG<12 was judged as moderate to severe MS (grade 3). MPG≥12 was judged as severe MS (grade 4).

**The detailed data of patients who didn’t receive operations**

Among the nine patients of UCMV patients, two asymptomatic patients in class I and one asymptomatic patient in class II were clinically followed up and found to have persistently severe (class I) and moderate (1/class I and 1/class II) MR. One patient with severe MR in the class I group did not undergo operation because of associated anomalies of aortic arch interruption and resistant pulmonary hypertension. The other five patients were all in the class III group, of whom two infants and one child unfortunately died of severe congestion of the pulmonary circulation before the operation could be performed; two children were followed up in the clinic and remained in NYHA functional II–III class because their parents were hesitant to provide consent for the operation.

Eleven patients in the control group did not undergo mitral valvuloplasty, of whom one infant with a hypoplastic MV died of cardiac arrest before the operation could be performed, and seven patients with moderate and three with severe MR were clinically followed up and remained in the NYHA functional II class.

**Online Table 2** **The classification of UCMV and Its related terms**

| **UCMV** | **Associated papillary muscle** | **Undifferentiated chorda group** | **MV dysfunction** | **Related terms** |
| --- | --- | --- | --- | --- |
| Class I | ALPM | ALCT | MR | Mitral hemi-arcade; Partial hammock MV; Parachute-like asymmetric MV |
| Class II | ALPM and PMPM | ALCT and PMCT | MR | Mitral arcade; Hammock MV |
| Class III | ALPM and PMPM | ALCT and PMCT | MS or MR+MS | Congenital typical MS; Mitral arcade; Hammock MV |

UCMV = undifferentiated chordae tendineae of the mitral valve, ALCT = chordae attached to the ALPM, PMPM = posteromedial papillary muscle, PMCT = chordae attached to the PMPM, MR = mitral regurgitation, MS = mitral stenosis, MV= mitral valve.

**Online Table 3. Mitral valve diagnostic parameters using echo and surgery**

| Variable | Sensitivity | Specificity | Accuracy | Kappa | Kappa P |
| --- | --- | --- | --- | --- | --- |
| Associated chorda group | - | - | - | 0.857 | <0.001 |
| ALPM | 100.00% | - | 100.00% | - | - |
| ALCT | - | - | - | 0.4 | <0.001 |
| PMPM | 91.50% | 92.90% | 92.00% | 0.831 | <0.001 |
| PMCT | - | - | - | 0.626 | <0.001 |

ALCT = anterolateral chordae tendineae, ALPM = antero-lateral papillary muscle, PMCT = posteromedial chordae tendineae, PMPM = posteromedial papillary muscle.

**Online Table 4. Adjusted characteristics of the UCMV and control groups**

|  | **Level** | **Overall** | **1** | **2** | **p** |
| --- | --- | --- | --- | --- | --- |
| n |  | **77** | **38.7** | **38.3** |  |
| **Baseline Characteristics** |  |  |  |  |  |
| Sex (%) | Male | 37.3 (48.5) | 19.3 (49.8) | 18.1 (47.1) | 0.788 |
|  | Female | 39.7 (51.5) | 19.4 (50.2) | 20.3 (52.9) |  |
| Age (median [IQR]) |  | 11.0 [6.0, 25.0] | 10.0 [7.0, 24.6] | 13.3 [5.0, 31.4] | 0.889 |
| Height (median [IQR]) |  | 73.0 [65.0, 90.1] | 71.0 [66.6, 83.6] | 75.0 [64.0, 91.4] | 0.862 |
| Weight (median [IQR]) |  | 8.2 [6.3, 11.7] | 8.2 [6.4, 10.8] | 8.3 [6.3, 12.0] | 0.898 |
| BSA (median [IQR]) |  | 0.4 [0.3, 0.5] | 0.4 [0.3, 0.5] | 0.4 [0.3, 0.6] | 0.832 |
| Cardiothoracic ratio (mean (SD)) |  | 0.6 (0.1) | 0.6 (0.1) | 0.6 (0.1) | 0.944 |
| Oxygen saturation (median [IQR]) |  | 98.5 [96.0, 100.0] | 98.8 [96.0, 100.0] | 98.0 [96.0, 100.0] | 0.513 |
| NYHA (%) | I | 26.2 (34.1) | 13.0 (33.6) | 13.2 (34.5) | 1 |
|  | II | 38.2 (49.7) | 19.3 (49.9) | 18.9 (49.4) |  |
|  | III | 11.0 (14.3) | 5.6 (14.5) | 5.4 (14.0) |  |
|  | IV | 1.5 (2.0) | 0.8 (2.0) | 0.8 (2.0) |  |
| MR grade (%) | 0 | 0.0 (0.0) | 0.0 (0.0) | 0.0 (0.0) | 0.959 |
|  | 1 | 0.0 (0.0) | 0.0 (0.0) | 0.0 (0.0) |  |
|  | 2 | 8.6 (11.2) | 4.2 (10.8) | 4.4 (11.5) |  |
|  | 3 | 17.3 (22.4) | 8.3 (21.4) | 9.0 (23.5) |  |
|  | 4 | 51.1 (66.4) | 26.2 (67.7) | 24.9 (65.0) |  |
| MS grade (%) | 0 | 73.7 (95.7) | 37.0 (95.7) | 36.7 (95.7) | 0.999 |
|  | 1 | 2.4 (3.1) | 1.2 (3.1) | 1.2 (3.0) |  |
|  | 2 | 1.0 (1.2) | 0.5 (1.2) | 0.5 (1.3) |  |
|  | 3 | 0.0 (0.0) | 0.0 (0.0) | 0.0 (0.0) |  |
|  | 4 | 0.0 (0.0) | 0.0 (0.0) | 0.0 (0.0) |  |
| Anomaly (%) | None | 17.2 (22.3) | 8.0 (20.8) | 9.2 (23.9) | 0.909 |
|  | Simple | 51.1 (66.3) | 26.4 (68.3) | 24.7 (64.4) |  |
|  | Complex | 8.7 (11.3) | 4.2 (10.9) | 4.5 (11.7) |  |
| Previous MV Op (%) | None | 71.0 (92.2) | 35.6 (92.0) | 35.4 (92.3) | 0.951 |
|  | Yes | 6.0 (7.8) | 3.1 (8.0) | 2.9 (7.7) |  |
| **Operative Data** |  |  |  |  |  |
| CPB time (median [IQR]) |  | 105.0 [86.5, 123.0] | 116.8 [94.4, 132.0] | 100.0 [77.8, 112.7] | 0.011 |
| ACC time (median [IQR]) |  | 73.0 [54.7, 85.6] | 79.0 [63.7, 90.0] | 66.5 [47.7, 77.0] | 0.007 |
| Posterior annuloplasty (%) | None | 5.3 (6.9) | 1.6 (4.2) | 3.7 (9.7) | 0.256 |
|  | Yes | 71.7 (93.1) | 37.1 (95.8) | 34.6 (90.3) |  |
| Leaflet plication (%) | None | 36.0 (46.8) | 12.0 (31.1) | 24.0 (62.6) | 0.002 |
|  | Yes | 41.0 (53.2) | 26.7 (68.9) | 14.3 (37.4) |  |
| Secondary chorda resection (%) | None | 43.2 (56.0) | 19.2 (49.7) | 23.9 (62.4) | 0.204 |
|  | Yes | 33.8 (44.0) | 19.4 (50.3) | 14.4 (37.6) |  |
| Papillary muscle splitting (%) | None | 45.3 (58.9) | 14.1 (36.6) | 31.2 (81.3) | <0.001 |
|  | Yes | 31.7 (41.1) | 24.5 (63.4) | 7.2 (18.7) |  |
| Chorda detachment (%) | None | 63.5 (82.4) | 31.0 (80.2) | 32.4 (84.6) | 0.584 |
|  | Yes | 13.5 (17.6) | 7.7 (19.8) | 5.9 (15.4) |  |
| Chorda shortening (%) | None | 66.5 (86.4) | 35.6 (92.0) | 30.9 (80.7) | 0.108 |
|  | Yes | 10.5 (13.6) | 3.1 (8.0) | 7.4 (19.3) |  |
| Leaflet patch augmentation (%) | None | 73.9 (96.0) | 35.9 (92.8) | 38.0 (99.2) | 0.015 |
|  | Yes | 3.1 (4.0) | 2.8 (7.2) | 0.3 (0.8) |  |
| Leaflet resection (%) | None | 75.0 (97.4) | 36.7 (94.8) | 38.3 (100.0) | 0.147 |
|  | Yes | 2.0 (2.6) | 2.0 (5.2) | 0.0 (0.0) |  |
| Leaflet cleft closure (%) | None | 69.0 (89.6) | 38.0 (98.1) | 31.1 (81.1) | 0.004 |
|  | Yes | 8.0 (10.4) | 0.7 (1.9) | 7.2 (18.9) |  |
| Supramitral ring resection (%) | None | 73.8 (95.8) | 36.3 (93.9) | 37.4 (97.7) | 0.339 |
|  | Yes | 3.2 (4.2) | 2.3 (6.1) | 0.9 (2.3) |  |
| **Outcomes** |  |  |  |  |  |
| AE (%) | None | 70.0 (90.9) | 34.6 (89.5) | 35.4 (92.4) | 0.584 |
|  | Yes | 7.0 (9.1) | 4.1 (10.5) | 2.9 (7.6) |  |
| AE MR (%) | None | 73.1 (95.0) | 36.4 (94.0) | 36.8 (96.0) | 0.658 |
|  | Yes | 3.9 (5.0) | 2.3 (6.0) | 1.5 (4.0) |  |
| AE MS (%) | None | 75.9 (98.6) | 38.0 (98.1) | 37.9 (99.0) | 0.619 |
|  | Yes | 1.1 (1.4) | 0.7 (1.9) | 0.4 (1.0) |  |
| AE MVR (%) | None | 75.4 (97.9) | 37.6 (97.3) | 37.7 (98.4) | 0.667 |
|  | Yes | 1.6 (2.1) | 1.0 (2.7) | 0.6 (1.6) |  |
| AE ReOp (%) | None | 74.6 (96.8) | 36.6 (94.7) | 37.9 (99.0) | 0.075 |
|  | Yes | 2.4 (3.2) | 2.1 (5.3) | 0.4 (1.0) |  |
| Repeated on-pump (%) | None | 70.2 (91.2) | 34.4 (89.1) | 35.8 (93.4) | 0.453 |
|  | Yes | 6.8 (8.8) | 4.2 (10.9) | 2.5 (6.6) |  |
| Ventilation time (median [IQR]) |  | 22.0 [9.0, 48.8] | 22.0 [9.0, 45.4] | 21.8 [8.0, 50.5] | 0.779 |
| ICU stay (median [IQR]) |  | 100.0 [63.9, 146.2] | 96.0 [45.2, 192.0] | 117.0 [88.4, 144.0] | 0.129 |
| Peritoneal dialysis (%) | None | 76.4 (99.2) | 38.2 (98.7) | 38.2 (99.8) | 0.085 |
|  | Yes | 0.6 (0.8) | 0.5 (1.3) | 0.1 (0.2) |  |
| FU time (median [IQR]) |  | 8.3 [2.5, 13.9] | 10.0 [3.2, 15.9] | 6.7 [1.5, 12.2] | 0.092 |

Results are given as the number (percent) or the mean ± SD or median (interquartile range). Simple anomaly including: ventricle septal defect, atrial septal defect; patent ductus arteriosus. Complex anomaly including: aortic valve stenosis, aortic arch interruption; coarctation of the aorta; sub-aortic valve stenosis; hypertrophic cardiomyopathy, Tetralogy of Fallot. UCMV = undifferentiated chordae tendineae of the mitral valve, MV= mitral valve, BSA = body surface area, SD = standard deviation, IQR = interquartile range, MR = mitral regurgitation, MS = mitral stenosis, NYHA = New York Heart Association, AE = adverse event, AE-MR = mitral regurgitation increase or severe mitral regurgitation, AE-MS = severe mitral stenosis, AE-MVR = mitral replacement, AE-ReOp = mitral re-operation, OR = operation, ALPM = antero-lateral papillary muscle, ALCT = chordae attached to the ALPM, PMPM = posteromedial papillary muscle, PMCT = chordae attached to the PMPM, ACC = aortic clamping, CPB = cardiopulmonary bypass, ICU = intensive care unit, FU = follow-up

**Online Figure 1 Normal and short chordae**

**a**. 2D echo shows normal ALCT and PMCT. **b**. 3D echo shows normal ALPM, ALCT, PMPM, and PMCT **c**. 2D echo shows short ALCT and PMCT and hypertrophic and elongated ALPM and PMPM. **d**. 3D echo shows short ALCT and PMCT and a fibre bridge (FB) below the anterior leaflet. 2D = two dimensional, 3D = three dimensional, ALPM = anterolateral papillary muscle, ALCT = chordae attached to the ALPM, PMPM = posteromedial papillary muscle, PMCT = chordae attached to the PMPM, FB = fibre bridge.

**Online Figure 2 PSM between the UCMV and control groups**

1. PSM with matching weights was used to balance differences in the baseline status between the UCMV and control groups. **b**. ‘love plot’ graphical display of covariate balance before and after adjustment. UCMV = undifferentiated chordae tendineae of the mitral valve, PSM = propensity score matching.

**Online Figure 3 ‘bare’ leaflet**

* ‘bare’ parts of the anterior leaflet.

**Online video legends**

**Online figure 1a**. Two demensional (2D) echo shows normal ALCT, ALPM and PMCT, PMPM.

**Online figure 1b**. three demensional (3D) echo shows normal ALCT, ALPM and PMCT, PMPM.

**Online figure 1c**. 2D echo shows short ALCT and PMCT.

**Online figure 1d**. 3D echo shows a fiber bridge (FB) below the anterior leaflet.

ALCT = chordae tendineae chordae tendineae attached to the anterolateral papillary muscle, ALPM=anterolateral papillary muscle , FB = fiber bridge, PMCT = chordae tendineae attached to the posteromedial papillary muscle，PMPM = posteromedial papillary muscle.
