# Supplementary figures and images for: Undifferentiated Chordae Tendineae of the Mitral Valve: Large Cohort Study of a Rare Mitral Malformation
Source: Front Cardiovasc Med. 2021 Jul 27;8:695536. doi: 10.3389/fcvm.2021.695536 (PMC8353112; doi:10.3389/fcvm.2021.695536)

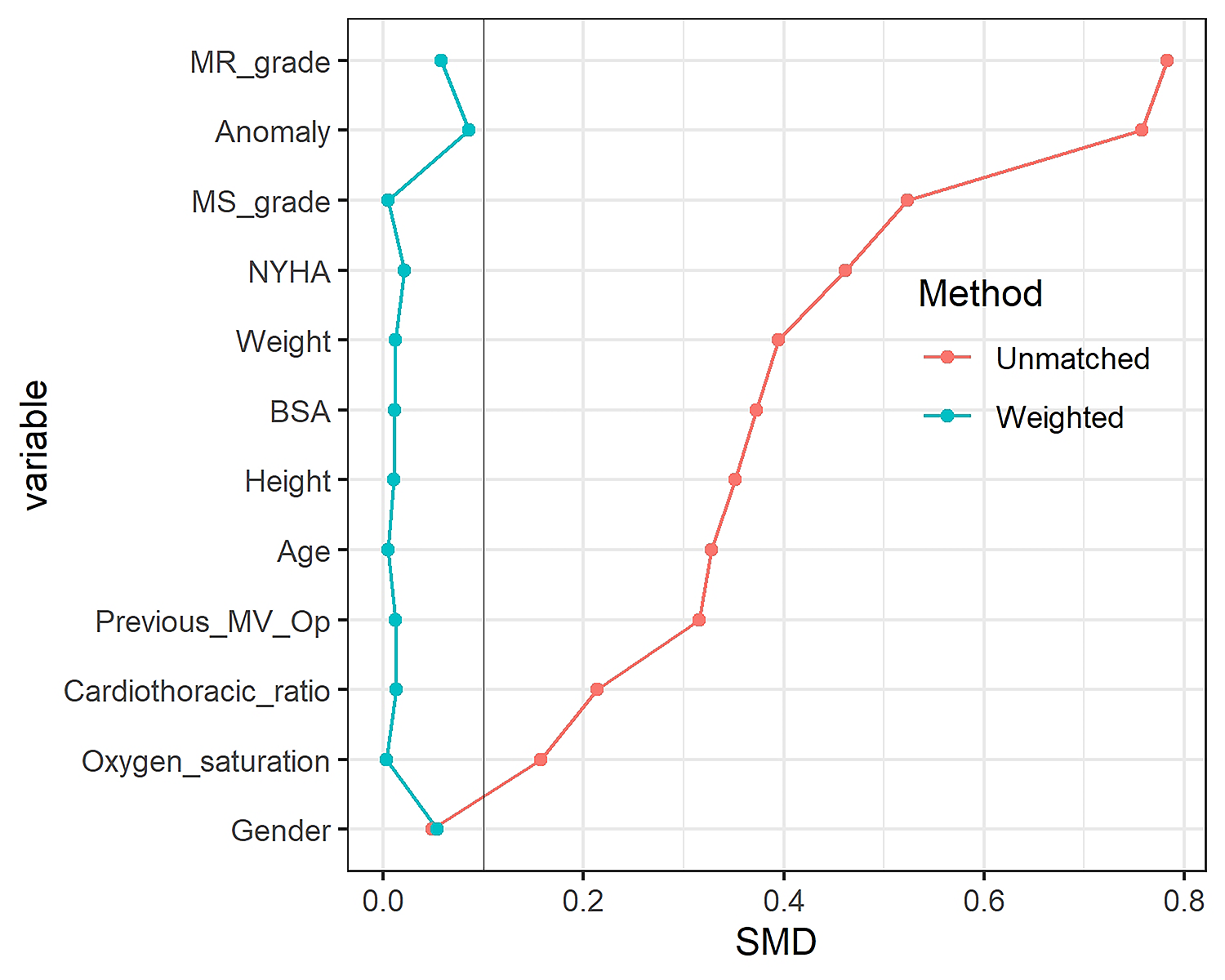

Supplement: Supplementary Figure 1. Class I: (b) — The colour Doppler of two-dimensional (2D) echo shows restricted severe mitral regurgitation. (c) 2D echo shows that the anterolateral chordae tendineae (ALCT) are absent and the anterolateral papillary muscle (ALPM) is hypertrophic and elongated and is directly connected with the anterior leaflet (AL). The posteromedial papillary muscle (PMPM) and chordae tendineae (PMCT) are relatively normal. (d) Three-dimensional (3D) echo shows the absent ALCT and the hypertrophic and elongated ALPM being directly attached to the AL, with the PMCT and PMPM being normal. (e) Surgical exploration shows that the ALPM is directly connected with the anterior commissure of the AL, and ALCT are absent. (f) Surgical exploration shows that the PMCT are relatively normal. Class II: (b) The colour Doppler of 2D echo shows restricted mitral regurgitation. (c) 2D echo shows that both the ALCT and PMCT are almost absent, and the ALPM and PMPM are hypertrophic and elongated and directly connected with the AL. (d) 3D echo shows the extremely hypertrophic and elongated ALPM and PMPM, which are directly connected with the AL. The ALCT and PMCT are absent. (e) Surgical exploration shows that the ALCT are absent, and the extremely hypertrophic ALPM are directly attached to the AL. (f) Surgical exploration shows that the PMCT are absent, and the extremely hypertrophic PMPM are directly connected with the AL. Class III: (b) The colour Doppler of 2D echo shows mitral stenosis plus mitral regurgitation. (c) 2D echo shows that both the ALCT and PMCT are absent. The ALPM and PMPM are hypertrophic and elongated and directly connected with the AL. (d) 3D zoom echo shows hypertrophic and elongated ALPM and PMPM, which are directly connected with the AL. The ALCT and PMCT are absent. (e) Surgical exploration shows the hypertrophic ALPM union with the AL. (f) Surgical exploration shows the hypertrophic PMPM union with the AL. [file Data_Sheet_2.ZIP › online figure/online figure2b.tif]

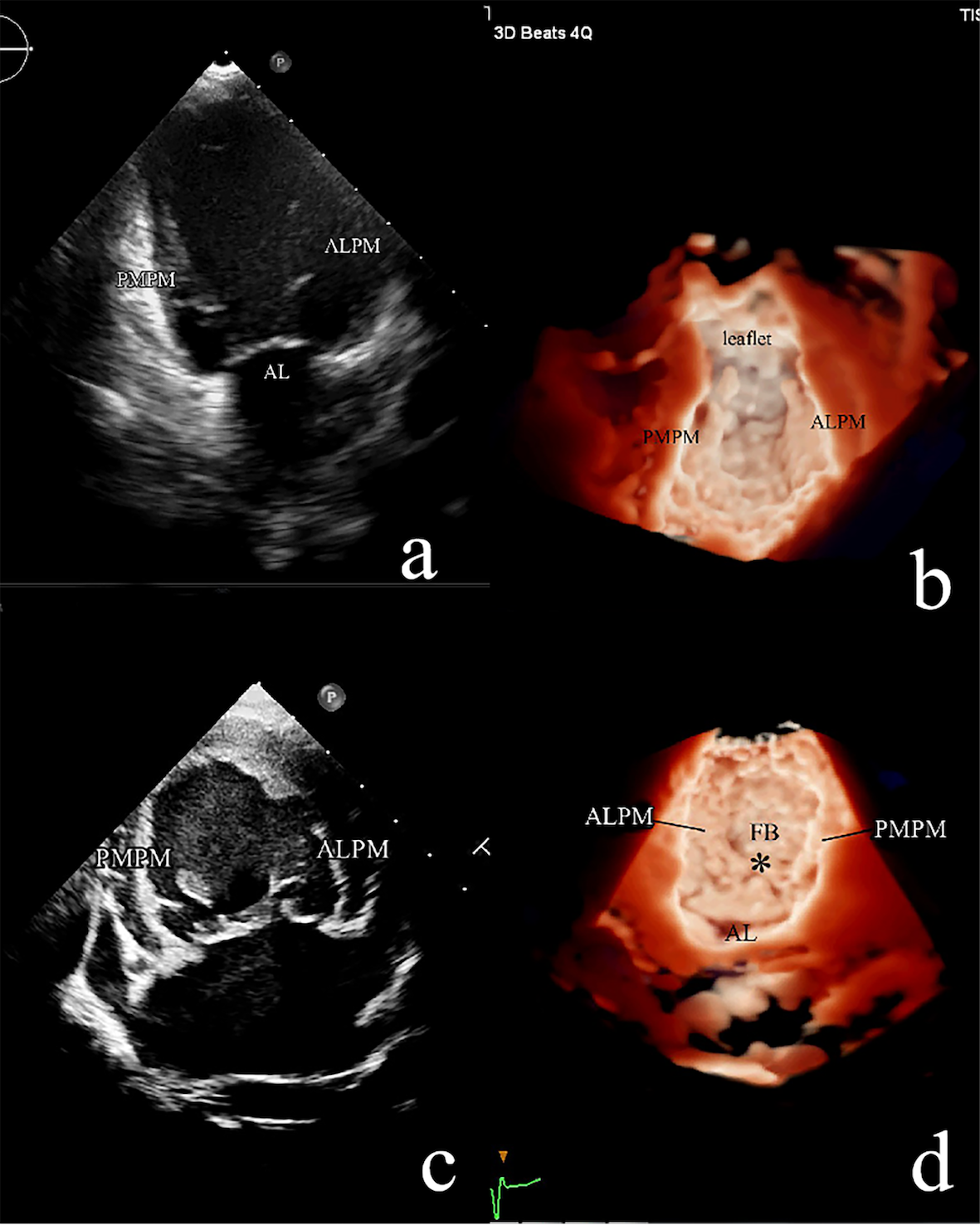

Supplement: Supplementary Figure 1. Class I: (b) — The colour Doppler of two-dimensional (2D) echo shows restricted severe mitral regurgitation. (c) 2D echo shows that the anterolateral chordae tendineae (ALCT) are absent and the anterolateral papillary muscle (ALPM) is hypertrophic and elongated and is directly connected with the anterior leaflet (AL). The posteromedial papillary muscle (PMPM) and chordae tendineae (PMCT) are relatively normal. (d) Three-dimensional (3D) echo shows the absent ALCT and the hypertrophic and elongated ALPM being directly attached to the AL, with the PMCT and PMPM being normal. (e) Surgical exploration shows that the ALPM is directly connected with the anterior commissure of the AL, and ALCT are absent. (f) Surgical exploration shows that the PMCT are relatively normal. Class II: (b) The colour Doppler of 2D echo shows restricted mitral regurgitation. (c) 2D echo shows that both the ALCT and PMCT are almost absent, and the ALPM and PMPM are hypertrophic and elongated and directly connected with the AL. (d) 3D echo shows the extremely hypertrophic and elongated ALPM and PMPM, which are directly connected with the AL. The ALCT and PMCT are absent. (e) Surgical exploration shows that the ALCT are absent, and the extremely hypertrophic ALPM are directly attached to the AL. (f) Surgical exploration shows that the PMCT are absent, and the extremely hypertrophic PMPM are directly connected with the AL. Class III: (b) The colour Doppler of 2D echo shows mitral stenosis plus mitral regurgitation. (c) 2D echo shows that both the ALCT and PMCT are absent. The ALPM and PMPM are hypertrophic and elongated and directly connected with the AL. (d) 3D zoom echo shows hypertrophic and elongated ALPM and PMPM, which are directly connected with the AL. The ALCT and PMCT are absent. (e) Surgical exploration shows the hypertrophic ALPM union with the AL. (f) Surgical exploration shows the hypertrophic PMPM union with the AL. [file Data_Sheet_2.ZIP › online figure/online figure1.tif]

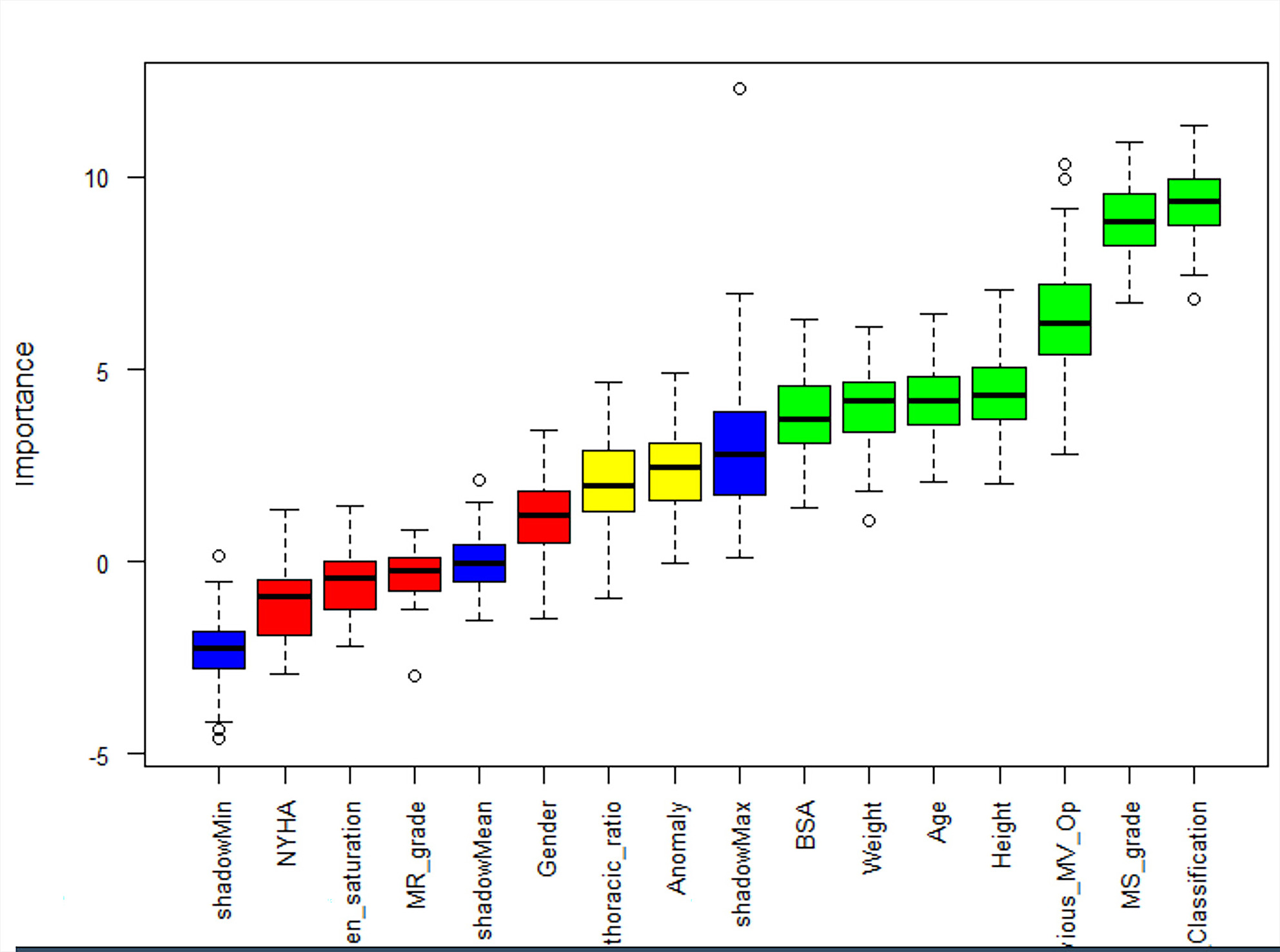

Supplement: Supplementary Figure 1. Class I: (b) — The colour Doppler of two-dimensional (2D) echo shows restricted severe mitral regurgitation. (c) 2D echo shows that the anterolateral chordae tendineae (ALCT) are absent and the anterolateral papillary muscle (ALPM) is hypertrophic and elongated and is directly connected with the anterior leaflet (AL). The posteromedial papillary muscle (PMPM) and chordae tendineae (PMCT) are relatively normal. (d) Three-dimensional (3D) echo shows the absent ALCT and the hypertrophic and elongated ALPM being directly attached to the AL, with the PMCT and PMPM being normal. (e) Surgical exploration shows that the ALPM is directly connected with the anterior commissure of the AL, and ALCT are absent. (f) Surgical exploration shows that the PMCT are relatively normal. Class II: (b) The colour Doppler of 2D echo shows restricted mitral regurgitation. (c) 2D echo shows that both the ALCT and PMCT are almost absent, and the ALPM and PMPM are hypertrophic and elongated and directly connected with the AL. (d) 3D echo shows the extremely hypertrophic and elongated ALPM and PMPM, which are directly connected with the AL. The ALCT and PMCT are absent. (e) Surgical exploration shows that the ALCT are absent, and the extremely hypertrophic ALPM are directly attached to the AL. (f) Surgical exploration shows that the PMCT are absent, and the extremely hypertrophic PMPM are directly connected with the AL. Class III: (b) The colour Doppler of 2D echo shows mitral stenosis plus mitral regurgitation. (c) 2D echo shows that both the ALCT and PMCT are absent. The ALPM and PMPM are hypertrophic and elongated and directly connected with the AL. (d) 3D zoom echo shows hypertrophic and elongated ALPM and PMPM, which are directly connected with the AL. The ALCT and PMCT are absent. (e) Surgical exploration shows the hypertrophic ALPM union with the AL. (f) Surgical exploration shows the hypertrophic PMPM union with the AL. [file Data_Sheet_2.ZIP › online figure/online figure2a.tif]

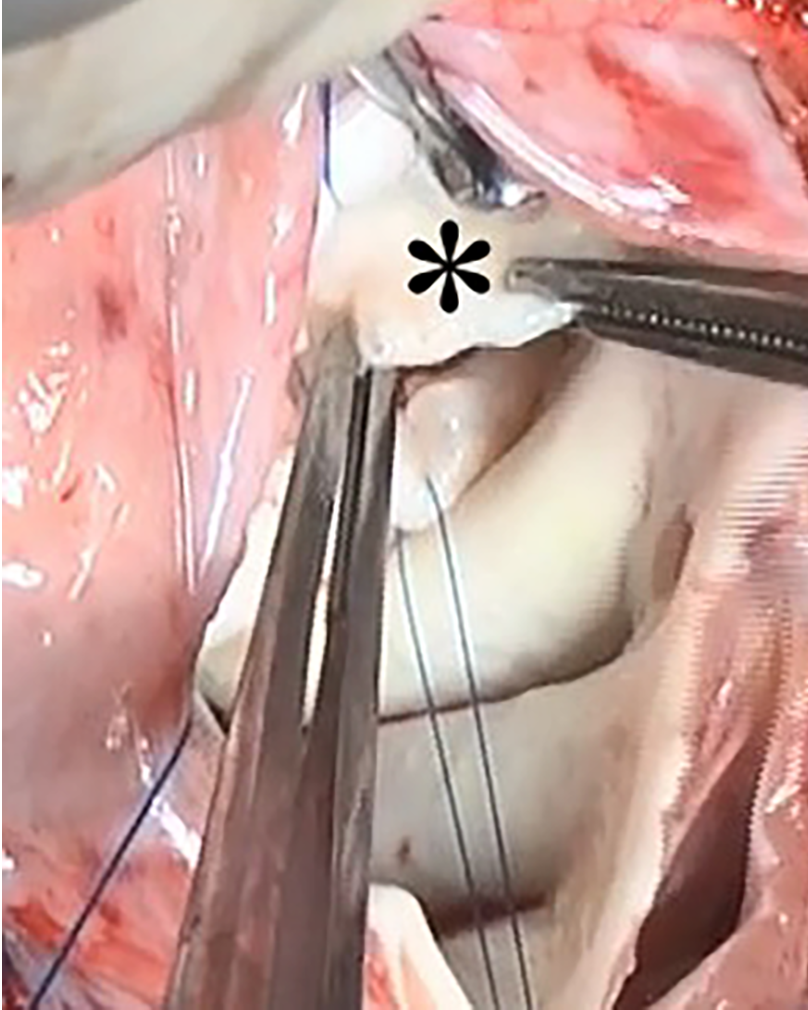

Supplement: Supplementary Figure 1. Class I: (b) — The colour Doppler of two-dimensional (2D) echo shows restricted severe mitral regurgitation. (c) 2D echo shows that the anterolateral chordae tendineae (ALCT) are absent and the anterolateral papillary muscle (ALPM) is hypertrophic and elongated and is directly connected with the anterior leaflet (AL). The posteromedial papillary muscle (PMPM) and chordae tendineae (PMCT) are relatively normal. (d) Three-dimensional (3D) echo shows the absent ALCT and the hypertrophic and elongated ALPM being directly attached to the AL, with the PMCT and PMPM being normal. (e) Surgical exploration shows that the ALPM is directly connected with the anterior commissure of the AL, and ALCT are absent. (f) Surgical exploration shows that the PMCT are relatively normal. Class II: (b) The colour Doppler of 2D echo shows restricted mitral regurgitation. (c) 2D echo shows that both the ALCT and PMCT are almost absent, and the ALPM and PMPM are hypertrophic and elongated and directly connected with the AL. (d) 3D echo shows the extremely hypertrophic and elongated ALPM and PMPM, which are directly connected with the AL. The ALCT and PMCT are absent. (e) Surgical exploration shows that the ALCT are absent, and the extremely hypertrophic ALPM are directly attached to the AL. (f) Surgical exploration shows that the PMCT are absent, and the extremely hypertrophic PMPM are directly connected with the AL. Class III: (b) The colour Doppler of 2D echo shows mitral stenosis plus mitral regurgitation. (c) 2D echo shows that both the ALCT and PMCT are absent. The ALPM and PMPM are hypertrophic and elongated and directly connected with the AL. (d) 3D zoom echo shows hypertrophic and elongated ALPM and PMPM, which are directly connected with the AL. The ALCT and PMCT are absent. (e) Surgical exploration shows the hypertrophic ALPM union with the AL. (f) Surgical exploration shows the hypertrophic PMPM union with the AL. [file Data_Sheet_2.ZIP › online figure/online figure3.tif]
